# Suicide ideation in older people: a qualitative review and Meta-aggregation of Asian studies

**DOI:** 10.3389/fpsyt.2023.1169820

**Published:** 2023-08-21

**Authors:** Martin Christensen, Hiu Yin Chan, Yuen Yi Chan, Ka Yee Cheng, Tsz Yan Cheung, Tsz Yan Li, Jia Ling Situ, Po Lam Tam, Cheuk Chi Tse, Haixia Ma

**Affiliations:** ^1^School of Nursing, Faculty of Health and Social Sciences, Hong Kong Polytechnic University, Hong Kong, China; ^2^Interdisciplinary Centre for Qualitative Research, School of Nursing, Hong Kong Polytechnic University, Hong Kong, China

**Keywords:** Asian, ideation, major depressive disorder, melancholy, older person, qualitative, self-harm, suicide

## Abstract

**Aims:**

To appraise and synthesize qualitative studies examining older Asian people’s experiences of suicidal ideation.

**Design:**

Qualitative review and meta-aggregation.

**Data sources:**

Four databases were accessed to retrieve papers published between 1990 and 2022 including the grey literature, hand-searching of reference lists of retrieved papers and key journals. The phenomenon of interest included participants older than 60 years old, must have experienced a form of suicidal ideation and/or an unsuccessful attempt, had actively thought about harming themselves and be of Asian ethnicity.

**Review methods:**

This review was conducted according to Consolidated Criteria for Reporting Qualitative Research and the Joanna Briggs Institute’s System for the Unified Management of the Assessment and Review of Information.

**Results:**

Of the 289 potential studies, seven papers met the inclusion criteria. Two synthesized findings resulted from this review–*The Suffering Situation: A Life without Meaning in Older Age* and *The Healing Situation: A Life Worth Living*. The experiences of older Asian people varied from feelings of loneliness, despair and isolation to wanting to live a fruitful life into old age.

**Conclusion:**

Suicidal ideation in the older person is a growing concern especially with the rise in suicide in this age group. Rising health care costs and erosion of traditional family values means that the older person views themselves as a burden. However, because of the limited number of qualitative studies from an Asian perspective it is difficult to ascertain the full extent of the issues surrounding suicide in older people.

## Introduction and background

Suicide in the elderly is becoming a major concern globally due to increasing social isolation, widowhood, poor physical and mental health, and rising healthcare costs (1). There is evidence suggesting that the incidence of suicide attempts is amplified when the older person has received a terminal diagnosis or a chronic disease that will become debilitating over time (2, 3). A form of self-harm suicide is defined as any act of “*…termination of an individual’s life, resulting directly from a negative or positive act of the victim himself, which he knows will produce this fatal result”*, [(4), p. 42] or more recently “*the human act of self-inflicted, self-intentional cessation*” [(5), p. 4]. However, it is important to differentiate between self-harm and suicide where they are two dissimilar behaviors, with the former seen as a major risk factor for the latter (6, 7). When compared with younger people, suicide in the older person differs only because of the increased risk of repetition and sociodemographic factors such as loneliness and being single strongly correlated with repetitive self-harm behaviors [NICE (8)]. When gender is considered, older Asian men are twice as likely to instigate suicidal activities than women in the same age group (9). In addition, older people tend to use a variety of methods with a significant level of lethality, for example, ingesting poisons, jumping from height or hanging (10, 11) with older men being more successful because of a stronger inclination to end their life (12, 13).

The substantial rise in the aging population across different countries has shown a persistent upward trend in suicide rates (14). Evidence from the Global Burden of Disease study suggests the global elderly suicide rate is nearly three times higher than the age-standardized suicide mortality across all ages, with some regions (China and Sub-Saharan Africa) having higher than age-standardized rates (13). For example, there has been a 300% increase in elderly suicides rates in South Korea, surpassing Japan (15) because of the upsurge of the elderly population. There may be number of reasons for this, yet the unpreparedness for an aging population and a changing societal environment (e.g., globalization) has meant a rapid change in the traditional extended family system for the worse (16), so much so that traditional family values that the elderly cherish, have been irreparably eroded. This is further compounded by those older couples without children or older individuals who never married especially where attending to the physical and mental support into older age becomes difficult if not impossible (13). Compared to Western countries, Asian countries have a higher elderly-to-general-population suicide ratio (10). However, it has been estimated that elderly Asian suicide rates should be lower compared to those in the west, thanks to the Asian culture of filial piety and venerating the elderly, whereas current rates reflect the opposite (17).

A lack of social and familial support in older age has been coined dissociative society-type elderly suicide and has been integral in understanding the social context in which older people derive experiences and importance (18, 19). The social fabric, important for the elderly, has become challenging because of increasing industrialization and urbanization especially in the community, and so it has led to increasingly levels of social isolation and in return increasing levels of depression (20). However, the levels of depression experienced are often underdiagnosed, services underutilized and lack of awareness means that repeat suicide attempts are becoming commonplace (20, 21). Qualitatively, the experiences of those engaging in suicidal behaviors often report feeling unfulfilled or a lack of self–worth, feeling lonely and helpless, feeling a burden both physically and financially to their children and often experience various forms of elder abuse (22–25). In summary:

▪ Suicide in the elderly is a growing mental health issue globally as a result of a number of key financial and social issues often associated with old age;
▪ The elderly suicide rate is nearly three times higher than age-standardized suicide mortality across all ages;
▪ Elderly suicide attempts are often repetitive with increasing lethality.

## Justification for the review

An initial search of four databases (the Cochrane Database of Systematic Reviews, the JBI Evidence Synthesis, MEDLINE and PROSPERO) was undertaken. As a result of this initial search there were no systematic reviews qualitative or quantitative currently being investigated on the topic from an Asian context. Indeed, much of the published literature focuses on cohort and control group studies using recognized scales such as suicidal intent scales in eliciting data concerning the more quantitative characteristics of self-harm. For example, one case–control study estimated the relative suicide risk from various physical illnesses with clinical depression, sex and age (3). Yet, there are very few published studies that had as its focus the lived experience of suicide with the majority of these undertaken within a western context (11). For instance, one study used a grounded theory approach to explore the process by which elderly Belgian people decide to attempt suicide (26). Moreover, qualitative studies centered on the Asian context are sparse. Little western studies can be transferable to an Asian context due to cultural variation. Therefore, this review aims to describe qualitatively older Asian people’s experiences of suicidal ideation.

### Aim and review question

To appraise and synthesize qualitative studies examining older Asian people’s experiences of suicidal ideation and behavior.

### Keywords

Suicide ideation, suicide attempt, Asian, older person, qualitative

### Inclusion and exclusion criteria: participants and phenomena of interest

For inclusion into this review, studies must have defined the participant groups as being older than 60 years old, must have experienced a form of direct suicidal ideation, they had ever harmed themselves in the past 12 months, had actively thought about harming themselves and be of Asian ethnicity. Studies were excluded if the participant group was younger than 60 years old and where quantitative including multiple/mixed-method approaches were used in data collection.

### Types of studies

This qualitative review considered research studies that focused on qualitative data only. These included, but were not limited to, designs typical of qualitative research such as phenomenology, grounded theory, ethnography, narrative enquiry and discourse analysis. In addition, each study described a qualitative account using recognized qualitative approaches to data collection. For example, face-to-face or focus group interviews of older Asian persons who have engaged in self-harm behaviors. For inclusion in the meta-aggregation, each study included data analysis that is consistent with qualitative research methods comprising inductive or deductive thematic and content analysis, or narrative inquiry. Papers were excluded if the individuals under investigation engaged in suicidal attempt behaviors that used surveys, Likert scales or other quantitative approaches, and these were not primary research studies and not written in English.

## Methods

This qualitative systematic review was conducted following the Consolidated Criteria for Reporting Qualitative Research (COREQ) [28]. Using the pneumonic SPIDER (**S**ample, **P**henomenon of **I**nterest, **D**esign, **E**valuation, **R**esearch Type) for qualitative research considered the following when developing the search strategy:

**S**“Older-person” OR” elderly”

**P** of **I**“suicide” “ideation”

**D**“interview” OR “focus group” OR “observation”

**E**“views” OR “experiences” OR “attitude” OR “perceptions” OR “feelings”

**R**“qualitative”

### Search strategy

Four databases (CINAHL, PubMed, Embase and Medline) were accessed to retrieve papers published between 1990 and 2022. This date range was chosen because during the initial literature searching four qualitative papers from the 1990s were found. These were excluded during preliminary screening because either the participants were less than 60 years of age or the study was undertaken in a western context and did not included Asian participants. For this review, we defined Asian to be those populations who live along the western Pacific Rim and not those associated with the Indian subcontinent (e.g., India, Pakistan, Sri Lanka or Bangladesh). All potential research studies were eligible for inclusion in this review, for example, conference reports. Searching for terms related to the key words involved Self-harm*, Suicide*, Asia* were paired with terms relating to the older person, elderly or aged and the research approach. Further literature searching related to older persons’ experiences of suicide used wildcard terms in order to offer further combinations to each of the search terms.

### Study selection

**Figure 1:**
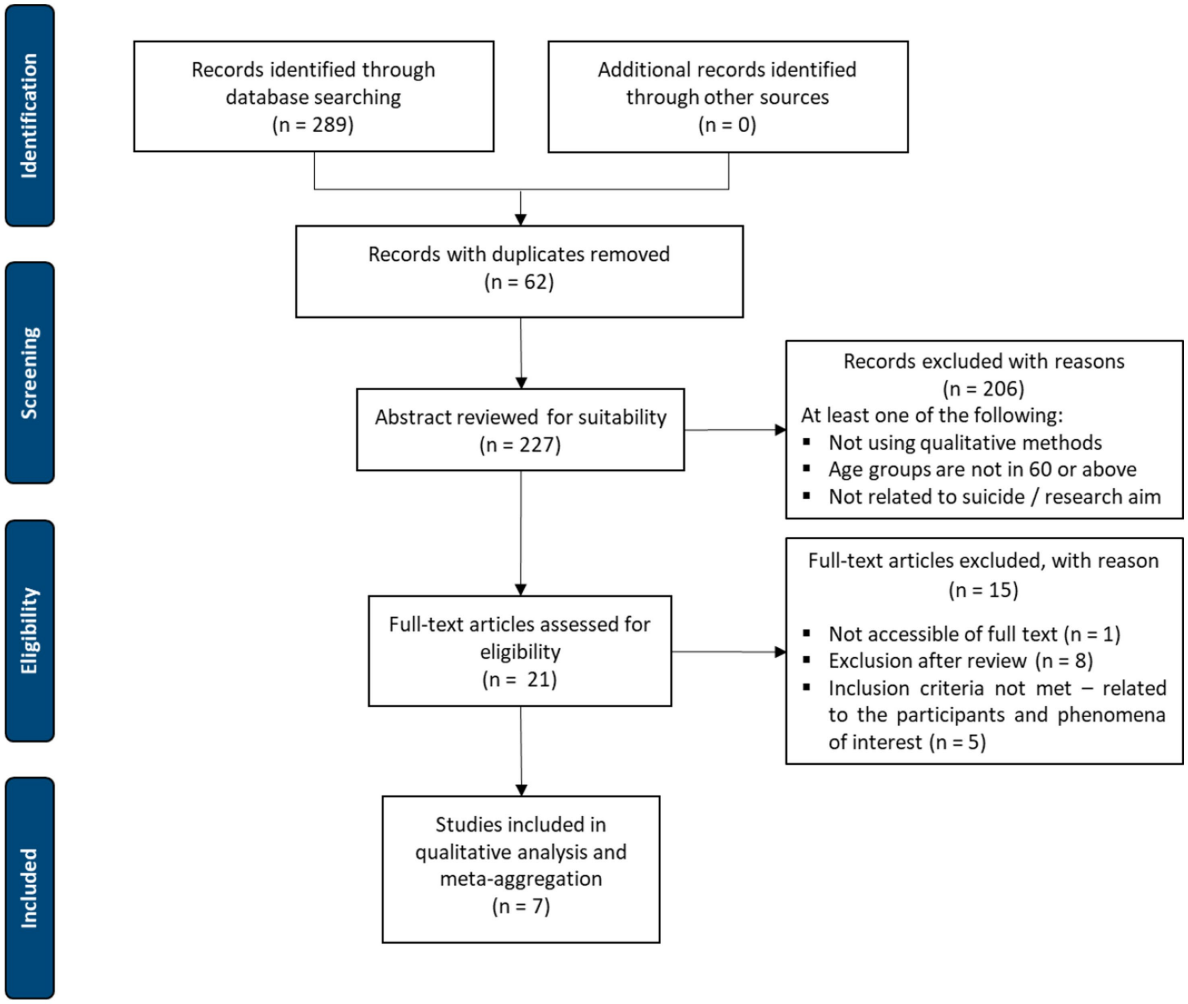
PRISMA flow chart of article selection, screening and assessment (29). A total of 289 articles were found. After screening and further refinement to the inclusion criteria, 7 were included in the final synthesis.

Following the search, all identified citations were collated and uploaded into an Excel spreadsheet with duplicates being removed. All eligible titles and abstracts identified from the search strategy were screened by two or more independent reviewers and assessed for relevance against the inclusion criteria. Those potentially relevant studies were retrieved in full text with their full citation details imported into the spreadsheet. The full text papers selected were then evaluated against the inclusion criteria by two or more independent reviewers. Reasons for exclusion at this point were recorded on the spreadsheet and included in the review as part of the PRISMA reporting. Disagreements arising between the reviewers at each stage of the study selection process were resolved through discussion, or if necessary, another reviewer. The Preferred Reporting Items for Systematic Reviews and Meta-analyses (PRISMA) flow diagram was used to document the results of the search strategy and the study inclusion process. A total of 289 articles were found and following screening and further refinement of the inclusion criteria, 7 were included in the final meta-aggregation synthesis (Figure 1).

### Assessment of methodological quality

Two independent reviewers, with expertise in qualitative research, critically appraised all eligible studies for methodological quality using the Joanna Briggs Institutes critical appraisal instrument for qualitative research based within the SUMARI programme. The methodological soundness of each of the eligible studies was assessed against philosophical and methodological domains. The 10 criteria making up the SUMARI appraisal involves assessing each paper against set criteria using either yes, no or unclear to ensure inter-rater trustworthiness (Table 1). Scores were calculated based on the number of yes responses, all papers were included in the final meta-aggregation. Cohen’s Kappa, was used to calculate the difference between observed agreement and agreement by chance which produced a coefficient of 0.91.

**Table 1:** Critical appraisal of included studies.

Study	Q1	Q2	Q3	Q4	Q5	Q6	Q7	Q8	Q9	Q10	Score
Huang et al. (23)	U	Y	Y	Y	Y	N	N	Y	Y	Y	7
Kim and Sok (19)	Y	Y	Y	Y	Y	U	Y	Y	Y	Y	9
Kim (20)	U	Y	U	Y	Y	U	U	Y	Y	Y	6
Ku et al. (32)	U	U	Y	Y	Y	N	N	Y	U	Y	4
Lee et al. (24)	U	Y	Y	Y	Y	N	N	Y	Y	Y	7
Lyndon et al.(33)	Y	Y	Y	Y	Y	N	N	Y	Y	Y	8
Tsai et al. (25)	U	Y	Y	Y	Y	N	N	Y	U	Y	6

Y, yes; N, no; U, unclear; JBI critical appraisal checklist for qualitative research.

Q1 = Is there congruity between the stated philosophical perspective and the research methodology?

Q2 = Is there congruity between the research methodology and the research question or objectives?

Q3 = Is there congruity between the research methodology and the methods used to collect data?

Q4 = Is there congruity between the research methodology and the representation and analysis of data?

Q5 = Is there congruity between the research methodology and the interpretation of results?

Q6 = Is there a statement locating the researcher culturally or theoretically?

Q7 = Is the influence of the researcher on the research, and vice-versa, addressed?

Q8 = Are participants, and their voices, adequately represented?

Q9 = Is the research ethical according to current criteria or, for recent studies, is there evidence of ethical approval by an appropriate body?

Q10 = Do the conclusions drawn in the research report flow from the analysis, or interpretation, of the data?

### Data extraction

Data extraction from the included studies was reviewed by two independent reviewers. The extracted data included specific details about the population under investigation, geographical location, study methodology (setting, theoretical framework, participants, sampling, data collection & data analysis) and the phenomena of interest relevant to the review objective, in this case suicide ideation (Table 2).

**Table 2:** Study characteristics.

Authors	Methods for data collection and analysis	Phenomena of interest	Context	Participant characteristics and sample size	Description of main results
Lyndon et al. (33), Malaysia	Individual interviews, Thematic Analysis	To examine the social risk factors that drive older people to have suicidal feelings or tendencies	Home environment	20 participants over the age of 60.	*Theme 1:* 5 primary risk factors identified**–**societal and cultural changes, lack of social support, conflict in religious beliefs, influence of economic instability and socio-economic status, and state of depression; *Theme 2:* changes that occur in the social structure of society as a result of modernization and industrialization; *Theme 3:* the impact of socio-economic status on the family; *Theme 4*: suicide was not solely attributed to mental illness but also due to the influence of the social environment.
Kim (20), South Korea	Individual interviews, Content Analysis	To describe life experiences after suicide attempts from the perspective of older Korean adults.	4 mental health clinics	35 elderly Koreans who had attempted suicide. Mean age 73	*Theme 1:* facing additional hardships including deteriorating physical health; *Theme 2:* experiencing higher levels of sadness and loneliness than before the suicide attempt; *Theme 3*: a deepening dependency on tranquilizers; *Theme 4:* seesawing between despair and faint hope.
Kim and Sok (19), South Korea	Individual interviews, Colaizzi’s approach to Descriptive Phenomenology	To understand the life experiences of elderly people who are living in long-term care hospitals and are afflicted with suicide ideation	3 long-term care hospitals	9 elderly individuals 65 years old or older with suicidal ideas	*Category 1*: Being a slave to one’s disease was how participants described their failing health after leaving hospital; *Category 2:* Sadness as a result of being far away from one’s family; *Category 3:* Vain care and consolation for me was described as the humiliation and shame of having to be cared for by others; *Category 4:* Continued life in a hospital, which feels like living abroad which was described as the feeling uncomfortable living with other people and feeling stuck in a different life that wasn’t their own; *Category 5:* Plunging life described the long-term hospital where losers live or those without a life anymore; *Category 6:* Moving toward the end of life is where participants described the futility of living a life bedridden; *Category 7:* Sad relief from death where participants described feeling cut off from the rest of the world and a life of being cared for.
Tsai et al. (25), Taiwan	Individual interviews, Colaizzi’s approach to Descriptive Phenomenology	To report a study of reasons for living among older male residents of veterans’ homes	Veterans Home	Participants were 36 residents who had expressed suicidal ideas in the past 6 months but had never shown suicidal behaviors	*Theme 1:* fear of death but being unable to take their own life; *Theme 2:* comparative improvement in health condition reduced the impetus to contemplate or attempt to self-harm/suicide; *Theme 3:* maintaining self-dignity; *Theme 4:* family-related concerns was described as feeling unable to kill themselves because of the loss experienced by others; *Theme 5:* concerns for staff often meant if they killed themselves then those cared for looking after them would get into trouble
Huang et al. (23), Taiwan	Individual interviews, Inductive Content Analysis	To discover the triggers for suicidal ideation, contributing psychological changes, and factors of adaptive response	Psychiatric outpatient department of one teaching hospital	32 participants ≥65 years, reported an intention to harm themselves within the past 6 months	*Theme 1:* Triggers for suicidal ideation including physical or health-related discomfort, negative feelings in response to experiencing a loss of respect or support from family members, personal conflicts or disputes, and painful memories; *Theme 2:* Psychological changes adding to suicidal ideation, which include feelings of loneliness and helplessness, and loss of self-worth; *Theme 3:* Preventive factors of continued suicidal ideation, which include social support, creating a fulfilling life, re-adjusting emotions, receiving effective treatment, religious beliefs, and focusing on family responsibilities.
Lee et al. (24), Taiwan	Individual interviews, Inductive Content Analysis	To explore triggers of suicide ideation among older first onset cases in psychiatric outpatients and their reasons for not executing suicide.	Psychiatric outpatients clinic	26 older psychiatric outpatients. Mean age 72	*Theme 1:* Reasons for suicidal thoughts**–**Illness and physical discomfort, conflicts with family members/friends, illness of family members, death of family members/friends, and loneliness; *Theme 2:* Participants’ reasons for not executing suicide**–**family members’ and friends’ support, receiving treatment, finding a way to shift their attention, fear of increasing pressure on one’ s children, religious beliefs, and not knowing how to execute suicide.
Ku et al. (32), Taiwan	Individual interviews, Inductive Thematic Analysis	To understand the suicide experiences, especially the triggers of suicide in institutionalized veterans	Veterans’ homes	19 older male participants living in a veterans’ residential care home. Mean age 80	*Theme 1:* Illness and pain. Participants described suffering from multiple illnesses and pain that interfered with their activities of daily life; *Theme 2:* Death of Close Relatives or Friends. Participants mentioned feelings of loss after the death of close relatives or friends. The grief process was very painful and endless, which induced their suicidal behaviors; *Theme 3:* Conflicts with Family Members. Participants described losing contact with or abandoned by family; *Theme 4:* Disputes with Friends or Workers. Participants described having arguments with their friends or workers before making suicidal attempts; *Theme 5:* Difficulty Adapting to Institutional Life. After moving into a strange place, they have to follow a highly structured daily schedule in the veterans’ home.

### Data synthesis

To determine the categories prior to meta-aggregation, each of the study’s findings were extracted together with illustrations of each finding to create a complete data set of all study findings. In order to determine credibility of the study findings, each finding and accompanying illustration was then deemed either unequivocal, credible or not supported (Table 3). The basis of the aggregation was to syntheses the results/findings into categories that have similarity in meaning (27). Thereafter, the categories were aggregated to make a single and inclusive set of findings that articulate qualitatively the experiences of suicidal ideation in older Asian people. All the findings from the studies reviewed were included for meta-aggregation and this produced two synthesized findings (Table 4).

**Table 3:** Example of meta-aggregative synthesis of findings into four categories to develop the synthesized find “the suffering situation: a life without meaning in older age”.

Finding	Category	Synthesized finding
Impulsive emotions/disputes or conflicts with others (U)	*Facing Conflict* (Describes situations and/or experiences of feeling disrespected by others)	*The Suffering Situation: A Life Without Meaning in Older Age* (Describes those negative feelings and thoughts as well as the psychophysiological decline that comes getting older)
Conflicts with Family Members (U)		
Disputes with Friends or Workers (U)		
Conflicts with family members/friends (U)		
Reasons for not executing suicide: Fear of increasing pressure on one’s children (U)		
Loss of respect and/or support from family (U)	*Disrespected* (Describes situations and/or experiences of feeling disrespected by others)	
Having More Sadness and Loneliness Than Before the Suicide Attempt (U)	*Loneliness, Helplessness and Despair* (Describes experiences where individuals identify with the purposelessness of their current situation)	
Loneliness (U)		
Sense of helplessness (U)		
Sadness as a result of being far away from one’s family (U)		
Continued life in a hospital, which feels like living abroad (C)		
Fear of death (U)		
Difficulty Adapting to Institutional Life (U)		
Plunging life (U)	*Feeling the need to attempt* (Describes situations where individuals can see no other alternative but to take their own life)	
Moving toward the end of life (U)		
Death of Close Relatives or Friends		
Illness of family members (U)		
Death of family members/friends (U)		
Triggers for suicidal ideation (U)	*The Emotional and Physical Pain* (Describes situations where individuals express depressive behaviors that coincide with the physical torment as a result of their age, includes those feelings of shame and embarrassment)	
Painful memories (U)		
Being a slave to one’s disease (U)		
Vain care and consolation for me (U)		
Illness and physical discomfort (U)		

U, unequivocal; C, credible (see text for description).

**Table 4:** ConQual summary of findings (32).

*Systematic Review Title:* A qualitative review of suicidal ideation in Asian older people *Population:* older Asian people who had or were contemplating suicide *Phenomena of Interest:* suicidal ideation *Context:* Older Asian persons >60 years of age				
Synthesized finding	Type of research	Dependability	Credibility	ConQual score
The suffering situation: a life without meaning in older age	Qualitative	Downgrade 1 level*	Downgrade 1 level**	Moderate
The healing situation: a life worth living	Qualitative	Downgrade 1 level*	Downgrade 1 level**	Moderate

* Downgraded one level due common dependability issues (all studies made no statement of locating the researcher culturally or theoretically).

** Downgraded one level due to a mix of unequivocal and equivocal findings.

### Assessing confidence in the findings

The final synthesized results were graded according to the ConQual approach (Tables 4 5). This approach allowed for establishing confidence (credibility and dependability) in the output of qualitative research synthesis. First, dependability was determined by the responses obtained from five questions (Q2-Q4, Q6, Q7 – see footer at Table 1 for explanation). Each of the studies was graded according to the number of “yes” responses present – 4-5 “yes” response (high) the finding remains unchanged, 2–3 “yes” responses the findings are downgraded one level (moderate) and 0–1 “yes” responses the findings are downgraded 2 levels (low; Table 4). Second, in determining credibility, the number of unequivocal verses credible findings was calculated. Unequivocal findings are defined as an illustration, significant statement or direct quote that is not open to scrutiny, whereas a credible finding is one that lacks a clear or definitive association and is consequently open to challenge (30). Therefore, ranking of unequivocal verses credible findings was based on the synthesized finding containing solely unequivocal findings (high) or a mixture of both unequivocal and credible (moderate, down-graded one level) or solely credible findings downgraded two levels (low). For those findings that contain neither unequivocal nor credible findings, they are further downgraded by 3–4 levels (very low). Based on the number of unequivocal (*n* = 38) verses credible (*n* = 3) findings meant that the level of credibility was downgraded one level. In reporting the overall findings of credibility and dependability a Summary of Findings was developed (Table 3) which, included the synthesized title, population under investigation, phenomena of interest and context for the specific review (31). For each synthesized finding identified from this review, the results were presented, together with the type of research approach used, the score for dependability (high to very low) and credibility (high to very low), and the overall ConQual score (high, moderate, low, very low). On the basis of this review the ConQual Summary of Findings produced a moderate rating of confidence in the synthesized findings.

**Table 5:** Ranking for dependability (32).

Congruity	Between method and question	Between methods and data collection	Between methods and data analysis	Researcher culturally and theoretically	Influence of the researcher	Grade
Huang et al. (23)	√	√	√	X	X	2–3
Kim and Sok (19)	√	√	√	√	X	2–3
Kim (20)	√	X	√	X	X	2–3
Ku et al. (32)	√	√	√	X	X	2–3
Lee et al. (24)	√	√	√	X	X	2–3
Lyndon et al. (33)	√	√	X	X	X	2–3
Tsai et al. (25)	√	√	√	X	X	2–3

Papers graded according to a mixture being present in study – “√” denotes present in paper and “X” was not present. The 5 questions above are those identified at the footer of Table 1.

## Results

### Study inclusion

The literature search of databases identified 289 potential articles written in English, for inclusion in this review, there were no papers identified through hand searching. After 62 duplicates were removed, the remaining 227 had their abstracts screened and reviewed, which excluded a further 206 papers that did not meet the inclusion criteria. The remaining 21 papers were retrieved for full text screening and a further 15 papers were excluded for a variety of reasons, including the inability to access the full text (*n* = 1), were excluded *via* SUMARI (*n* = 8) and the remaining five did not meet the inclusion criteria (Figure 1). The final seven papers were assessed for methodological quality and after discussion were included in this qualitative synthesis (Table 1).

### Methodological quality

The methodological quality of the seven papers using the JBI critical appraisal checklist for qualitative research and included for this qualitative synthesis ranged from high quality scores 9/10 (1 study), 8/10 (1 study) to moderate scores 7/10 (2 studies), 6/10 (2 studies), and low scores 4/10 (1 study; Table 1). Despite the single low score, after discussion it was agreed that this study should be included in the synthesis because of the level of detail in the participant voices. All of the papers were either unable or it was unclear as to the researchers influence on the study or where the researcher was located culturally or theoretically (Q6 & Q7). In five studies, the criteria for establishing congruity between the philosophical perspective and the research methodology (Q1) was unclear with the remaining two studies adopting a descriptive phenomenological approach. All papers were able to clearly articulate the congruity between the research method and data analysis (Q4), the research method and interpretation of the results (Q5) and the participants voices were represented (Q8).

### Characteristics of included studies

This qualitative review considered qualitative studies, which focused on suicidal ideation in older Asian people from 1990 to 2022. The studies identified were published between 2009 and 2021. There were seven studies conducted in three countries, four from Taiwan (23–25, 32), two from South Korea (19, 20) and one from Malaysia (33). The total sample size was 177 participants all aged over 60 years of age. The settings varied between studies with two being conducted in veterans homes (25, 32), three in psychiatric out-patient clinics (20, 23, 24), one in a long term care hospital (19) and one in the home environment (33). All of the studies included used a variety or recognized qualitative research designs to data collection and data analysis – two used descriptive phenomenology (19, 25), three used content analysis (20, 23, 24) and two used thematic analysis (32, 33). Individual interviews were the preferred approach to data collection in all studies (Table 3).

### Review findings

#### Synthesized finding–the suffering situation: a life without meaning in older age

In this synthesized finding “The Suffering Situation: A Life without Meaning in older age,” older Asian people described their life as painful and full of suffering. They lost the sense of purpose to continue living a meaningful life with the inescapable “black cloud” of despair and despondency that pervaded their thinking. Attempting suicide after a prolonged period of contemplation was seen as the only means of relief, allowing them in most cases to finally feel free from the suffering they were experiencing. There was a range of factors contributing to suicidal ideation in their older age. Both physical and/or psychological pain was viewed as directly contributing to the misery experienced in their later life. It was those feelings of loneliness, of hopelessness and helplessness that were significant drivers to suicidal ideation. Moreover, for the participants, it was the loss of important social support networks, not feeling or being respected, and the humiliation and shame of having to be physically exposed to carers were additional sources of resentment and ill feelings. The categories *Facing Conflict, Disrespected, Loneliness, Helplessness and Despair, Feeling the Need to Attempt*, and *The Emotional and Physical Pain* provides some insights as to the challenges that the elderly were facing in their later life, which unfortunately set them down a path of suicidal ideation and behavior.

### Facing conflict and feeling disrespected

The elderly often reported that prolonged conflict with family and friends led them to feeling isolated and alone and as such frequently increased those thoughts of suicidal ideation. The participants reported the repeated conflicts arose when they felt the family neglected their needs or undervalued them often because of their increasing functional decline both physical and mentally (20, 23–25). This increased their feelings of being seen as a burden or feeling worthless, which then led to a spiral of depression. This was exacerbated when their family or friends did not treat them with respect, which most viewed as a breakdown in the traditional family values (filial piety) (25, 32) and the growing trend of unfriendly communities. For example, one older male participant experienced first-hand the dismissive and effusive rudeness from his son when simply asking to see his grandson, which to him evoked feelings of being unimportant, unloved, and disrespected (32). When these conflicts were not handled well, older people generally developed various negative emotions and employed avoidance behaviors as a way of coping. As a result, they lost a sense of perspective especially around the meaning of living simply because the biggest part of their lives, their family, no longer seemed to care about or care for them (24). For example, one participant spoke of feeling betrayed and treated unfairly by friends and therefore decided that one way to “prove his innocence” was to attempt suicide (32). These experiences may be viewed as a collection of uncaring behaviors on the part of family and friends, yet for the older person experiencing them it creates a stressful situation one in which leaves them feeling angry and scared (33). Moreover, it is apparent that older people in this context often cannot find the proper way to effectively communicate and/or solve the problems they experience. Therefore, in order to seek attention and prove their self-worth, they may choose impulsive behaviors such as a suicide attempt to express their anger toward their closest ones (23).

Older people attempted to maintain their self-dignity by taking part in self-harming behaviors that sometimes resulted in a suicide attempt (23). This was often as a result of experiencing feelings of disrespect and especially disrespect by their own children, which was described as being heart-breaking (19). One participant expressed disappointment toward his “ungrateful sons”, who shouted at him repeatedly (24), while another suffered chest discomfort when his children simply ignored his opinion and were belligerent toward him, which increased his feelings of being seen as insignificant (23). Such was the physical and emotional pain these older people experienced and the constant reminder their lives were meaningless, that many felt the only solution was suicide (20, 23). One of the contributing factors to increasing suicidal thoughts, apart from the ungracious children’s behavior, was often attributed to being left out or not being a part of their children’s hectic family life, if anything just to feel included. Importantly, parents from traditional Asian families (based on Confucianism) believe that raising a child is an “insurance for aging”, in other words the time and effort devoted to raising them is expected to be paid off in later life as caring duties (24). Yet, in most cases, the children rarely visited their parents and for the parents this was seen as a violation of the traditional values associated with “filial piety” (19, 23, 32). It was, as they called it, an abandonment of the old traditions by their grown children that participants describe as a growing desperation, especially how they would be cared for as they got older. One older woman described her increasingly heavy reliance on her children to help with activities of daily living, but because of the “high time cost” associated with the type of care she needed, she was considered a burden by them. Moreover, she went onto state that she would rather voluntarily opt to being admitted to residential aged care facility than to continuing experience, what she describes as, the unbearable negligence and disrespectfulness of her children (33). These experiences of disrespect and impertinence were not isolated incidences, but importantly served as triggers to increased thoughts and at times acts of suicide simply because the older person’s presence was seen as unimportant and/or burdensome. Yet, in some cases this inevitably brought about an often irreversible and unfortunate loss to human life.

### Loneliness, helplessness, and despair

In this category, some older people experienced significant periods of low mood and affect because of their increased feelings of loneliness, helplessness, and despair brought on by increasing social isolation (20, 23). In particular, it was the loss of company that was one of the causes that aggravated feelings of loneliness, especially the loss of loved ones or close companions. Not only did this increase the sense of being alone, but also feeling lonely especially when grown up children had left the family home and were too busy to visit (19). One older man stated that he was afraid of being alone, particularly after his son moved out and the house was now empty (24). Being alone was one of the immediate reasons for loneliness and this was amplified when the older person lacked any sort of family support and therefore felt abandoned, which unfortunately reinforced those feelings of helplessness (33). Some of the participants were quick to point out the challenges they experienced when attempting to raise concerns about feeling and being alone with relatives who could neither understand nor be in a position to discuss these concerns with them. In most cases, they were simply unable to find someone, anyone to listen to them and comprehend the hardships they experienced, the pain from physical illness as well as dealing with the everyday stress of being physical unwell (20). Yet, they still needed to tolerate the pain every day and perform activities of daily living independently under trying circumstances. As a result, their quality of life also gradually declined and with it the overwhelming sense of helplessness due to what they perceived as their continual suffering as well as the inability to find help (20, 24, 32). This then created a scenario of de-socialization, which further aggravated their suffering. Some participants discharged from hospital to residential aged care especially after prolonged hospitalization, brought with it different challenges including adapting to a life of institutionalized living (25, 33). Despite living among others in similar circumstances, this often produced an increased sense of loneliness, helplessness and despair, which brought about an increase in suicidal thoughts, some seeing this as the only practical and necessary solution to ending their suffering (19).

### Feeling the need to attempt

There may be many reasons the older person decides to attempt suicide. Yet, the most common reason participants report is to end their suffering from physical illness or pain (20). Worsening chronic disease and increasing pain constantly impeded their daily lives (23). This often led to feelings of desperation and desolation with some driven to suicide ideation and behaviors as a means to ending the pain. Their long-term suffering had also negatively affected their ability to conduct activities of daily living, and as a result, the participants became increasingly more dependent on others, such as their children or partners. Some felt ashamed and embarrassed, especially when others needed to provide intimate care, which caused a heightened sense incapability and powerlessness (19, 25). As a result, this meant a declining sense of self-worth and motivation, which would significantly limit their capacity to perform even the simplest of tasks, such as bathing (25). There was even an increased sense of guilt because of the mounting cost of caring for them, especially health care costs, which some reported their families could ill afford (24, 33). This guilt almost always led to thoughts about suicide, more so when family members felt obligated to care for their elderly parents, often not out of filial duty but because of “saving face” with friends, neighbors or other family members (20). Sacrificing time and money to take care of them meant for most of the participants a need to end the unnecessary burden of looking after them (24) and therefore, suicide became a viable option or some suggested a form of self-euthanasia (19).

### The emotional and physical pain

The reciprocal action between physical and psychological health created a cycle of suffering for the participants. Some felt that the “torture” of physical illness influenced their emotions significantly (19). Some participants were increasingly worried and saddened as they faced numerous and worsening chronic health problems such as stroke and diabetes (20, 32). They regarded this as a form of “endless suffering”, especially with the need to take medication for the rest of their life and the additional financial cost this would incur (24). Therefore, to alleviate the “cycle of suffering”, many chose to end their lives. In some cases, participants felt that sustaining one’s life with assistance brings with it an emotional hurt. For instance, one participant spoke of the worst and most embarrassing experience was when his diaper was being changed (19). Another older man spoke of the humiliation and shame when told by his caregiver that he will experience increasing incontinence (19). The feeling of helplessness associated with not being able to complete essential daily activities was particularly overwhelming for some of the participants. For some, this resulted in flashbacks to their childhood and even echoed a series of unpleasant life events that happened in their younger days, days of hunger and poverty (20, 32). One participant spoke of his current illnesses as an accumulation of suffering throughout his life especially the difficulties and challenges he experienced as a younger man (20). For many the consolation of ending their lives brought with it a sense of relief, relief from the pain, the humiliation, shame and embarrassment of being cared for (19).

### Synthesized finding 2–the healing situation: a life worth living

In this synthesized finding The Healing Situation: A Life worth Living, older Asian people described those feelings associated with wanting to live, that is there is a fulfilling creative side to being an older person despite the perceived hardships that they may have experienced. There was the realization that the family support network, which was not always obvious to them, appeared to provide a greater source of comfort and relief. The categories, which align with this finding, encapsulate what was important for these older Asian people especially around re-establishing and strengthening support networks, but also the responsibility of what is they were hoping to achieve – an ending to their pain and suffering whatever that motivating factor may have been. The categories *Seeing the Benefits of Living, Returning from the Brink* and *Seeking Solace and Comfort* certainly gives testament to the strength of these older Asian people’s spirit when they realized the challenges that of being an older person can be overcome.

### Seeing the benefits of living

The first category to emerge from the synthesized finding centers on those descriptive experiences where the participants from the studies reviewed realized that there is a life worth living, one in which saw them re-adjusting their emotions more positively, focusing on their responsibilities to family and friends and maintaining a sense of self-dignity were important considerations (20, 23, 25). Some who were clearly distressed at their current health status (a cancer diagnosis or living with chronic disease) became less concerned with taking their own lives and the difficulties and upset that this would install and instead focused on how to improve their mental and physical wellbeing (24, 25). For example, one older person spoke of mobility issues associated with having severe osteoarthritis, which significantly impacted on his mental health to the point that suicide seemed the only way to relieve him of the pain (32). However, having being prescribed newer medications and rehabilitative treatment saw a dramatic improvement in his mobility and consequent mental health (20, 24, 32). Others spoke of the being told of their importance as a guiding figure in the family unit (24, 25), something that had gotten lost over time as children had grown up and left home, which increased their sense of isolation (19). One man describes feeling needed and wanted by his family providing him the strength to keep going or the realization at being informed by their wife that she wants him to live and for that reason wanting to live longer with her (24). Such was the emotiveness of these comments that many realized that this enabled them to re-adjust their thinking in a more positive light and therefore remain focused on their future (25).

### Returning from the brink

Having made an attempt to take their own life or in some case not knowing how to commit suicide, some older people ironically realized that the time is not right in making a serious attempt (23, 25). In some cases, this was in response to the attempt at suicide being more painful than that experienced with their current situation, for example not being able to simply drink water because of the extreme pain experienced after consuming pesticides or poison which had seriously damaged internal organs (20). In other cases, older peoples despair worsened as the realization that a failed attempt had brought actually worsened their problems as opposed to alleviating them (20, 24). While this may be seen as a “cry for help” for these older people it was a mixture of finally seeing the futility of the attempt or in other cases receiving treatment in the form of anti-depressants that instilled a sense of returning from the brink. Despite feeling the “time was not right” after seeking help any thought of suicide was drastically reduced because a combination of medications (23, 24) and being mindful of the potential outcomes of what a failed attempt would bring.

### Seeking solace and comfort

In this category, individuals adopted divergent behaviors to reduce self-harming thoughts. Concerns for others, seeking solace in religion or finding ways to shift their attention away from negative thoughts that had previously prompted ideation or at worst an attempt (33). For some, religion played a significant role in discouraging anxious thoughts that would often precede self-harm ideation (23–25). Meditation, prayer or simply being present in the temple often had the desired effect of reducing any idea of self-harm, even the simple act of reciting scripture was enough to divert thoughts and induce a sense of spiritual calmness and peace (23, 24). For others the family unit provide unique opportunities to avert negative thoughts by being aware of the void in the older persons life, either by the death of a spouse or through divorce and therefore provided other avenues for the older person to focus their attention such as grand-parenting duties (23–25). Yet for those living in residential aged care, the sad reality of their current life was offset by the kindness and care they received from the staff charged with caring for them. Some spoke of feeling grateful at being allowed to live a full and content life up until the end (19, 20).

## Discussion

The aim of this qualitative review was to describe self-harm ideation and self-harming behaviors in the older Asian population. The findings from this review indicate that self-harm is complex and multifactorial and often encompasses a suicide attempt, in some cases multiple attempts. Those precipitating factors that often precede a self-harming episode (including a suicide attempt) range from physical and emotional pain and stress to incidences of feeling disrespected, lonely, helpless and desperate. While some precipitating factors such as feeling disrespected by family members and the physical suffering associated with chronic illness were deemed by the older person as being uncontrollable, these were the most common experiences described. Yet, it was those feelings of loneliness, usually resulting from living alone and/or bereavement, together with helplessness and despair, which would inevitably trigger self-harming behaviors. It was those feelings of loss are what Li et al. (34) characterize as a “lost theory,” which suggests that aging is exemplified by continuous loss, including loss of one’s health, one’s social role, one’s relatives and friends and one’s life goals. This theory also supports the idea that loss for older people serves as a major crux for desperation ideation, which then leads into depression, social isolation and loneliness.

Moreover, the findings from this review also identified that the majority of Asian older people were mired in, what seemed like endless, suffering situations because of the loneliness, helplessness, and despair they experienced. It was these negative feelings that then fueled thoughts of suicide. Similar studies have also indicated that loneliness and isolation have a direct impact on aggravating suicide ideation and behavior and also positively correlates with a suicide attempt (6, 35–37). Interestingly, Troya et al. (7) also found that older people from their study viewed a suicide attempt as a “cry for help”, a means to rid themselves of the loneliness that comes with bereavement. Likewise, older people often felt that suicidal ideation was more frequently a coping mechanism than a suicidal expression, simply because they were unable to find alternative coping mechanisms to deal with adverse events and stressors. Therefore, the elderly believe that suicide is the most singular way to express these repressed feelings (7).

Yet, consumed within the tradition of filial piety, older people were often conflicted/confused between the conventional wisdom of venerating the elderly, the expectation of being cared for by children in old age and what they now experienced. In recent years, Chinese older people, for example define the concept of filial piety based on whether their children can practice the traditional values of raising children to protect against old age (38). The conflict or confusion often evolves when their children either refuse to care for their elderly parents or the parents are victims of elder abuse at the hands of their children, which creates an environment of distrust and disrespect. The resulting family conflict produced a sense of insignificance, betrayal and neglect on the part of the children (39). Moreover, it is thought the implementation of the “One-child policy” brought with it a significant structural change to the Chinese family (4-grandparents; 2-parents; 1-child). This resulted in the expense of caring for older family members increasing significantly, which meant the financial burden placed upon one child becomes untenable or impossible. Therefore, the pervading belief among older people is that as the cost of care increases, the willingness to care for them becomes increasingly less so. Unfortunately, this also brings the threat of financial insecurity to a threat of old-age security (40). This is also evident where globalization has exacerbated the “empty nest” syndrome, where grown-up children leave for other cities or indeed other countries (34).

This review also reports similar findings from those in a western context where emotional and physical pain experienced by older people can become a precursor to suicidal ideation. For example, Sousa et al.’s (41) integrative review of suicide risk in the elderly, found that chronic degenerative disease made up 61% of the physical ailments that beset the older person with over 30% of older people experiencing increased feelings of loneliness as a result. Likewise, in examining the mediators between physical pain, loneliness, social integration and suicide, Lutzman et al. (42) suggested that physical pain alone would not directly lead to suicidal ideation. However, they report that physical pain increases restrictions on activities of daily living leading to deliberate or enforced social isolation. Indeed, Wang et al.’s (43) study of Asian older immigrants living in New Zealand, having experienced similar mobility and self-care issues as a result of acute physical pain not only led them to becoming increasingly dependent on family assistance for essential care, but this also decreased their sense autonomy and increased feelings of being a burden. Therefore, the emotional pain brought on by social isolation combined with the physical pain of worsening chronic disease becomes a vicious cycle of loneliness, isolation and depression. Unfortunately, this can perpetuate and intensify suicidal thoughts (22).

However, this review also identified that despite some of the difficulties experienced by some older Asian people, like those mentioned above there were some older people who could find a sense of purpose for living into their old age. Being considered a guiding figure in the family, being told how important they were by spouses, or being prescribed newer medications that helped them see more clearly and view their lives more positively were some examples of purposeful living. This sense of positive living was echoed in Gutierrez et al.’s (44) study, which suggested that older adults initially construct new meanings from situations that may generate suicidal behaviors – loneliness, social isolation, and depression. Yet, along with these new meanings also comes a renewed sense of control over the own destiny suggesting that older people are capable of “coming out” of isolation and dispelling those feelings of worthlessness. This phenomenon is what Flett and Heisel (45) refer to as “mattering”. Flett and Heisel (45) suggested that retirement can be a significant precursor for older people not feeling mattered or being bothered about – a sense of unimportance. This sense of worthlessness or unimportance may arise because of declining social (friend’s dying or declining cognitive function) and productive activities when older people retire. This review along with others found that older people who never or rarely felt useful and important (not mattering) have a greater tendency toward self-ham due to poor psychological well-being, social isolation, and maladaptive health behaviors (46). Mattering also plays an important role in influencing the association between physical illness and loneliness among older people (47). In contrast, older adults who perceived they mattered stated of having a greater sense of their purpose in their lives, overall wellness and lower vulnerability to feelings of depression (48). In addition, the outcomes of a systematic review undertaken by AshaRani et al. (49), found that social relationships and mattering are significantly interrelated where participation in social or family relationships helps older people in developing a sense of belonging, being needed and respected.

### Review limitation

This study has several limitations, in which the conclusions drawn are from a small sample of qualitative studies reported in English may not be generalizable/transferable for all late-life Asian people. First, studies in other Asian languages, such as Chinese, Korean and Japanese were excluded to minimize translation errors and misinterpretation. This limitation also affects the diversity of the study population. Among the seven studies reviewed, four studies were conducted in Taiwanese elderly, two of them explored the experiences of the elderly in South Korea and one from Malaysia and as a result cultural difference may advertently influence the participants’ attitudes or beliefs regarding suicide and are therefore, responses may not be uniform throughout the region. Second, selection bias of the study participants exists in the majority of studies reviewed, so that the perception of emotions may differ from each other under the influence of various living environments, financial situations and local cultures. Third, older people who had made a suicide attempt but were unknown to public health services were not included in the sampling and the most significant being the exclusion of those participants with a mental health issue. While mental illness in older people is difficult to positively correlate with suicide ideation or behaviors, the relationship between them, however, cannot be ruled out entirely. It is, therefore, difficult to classify whether suicidal behavior is rooted merely in the “sorrows” of the elderly, or it is attributable to mental health issues when this criterion is not fully reviewed. Therefore, what this review has established is that:

▪ Increasing loneliness, social isolation, elder abuse and financial insecurity are major factors in a suicidal attempt;
▪ The breakdown in traditional family values (filial piety) and venerating the elderly is a major precursor to suicidal ideation;
▪ Shame and embarrassment individually and dishonoring the family (saving face) in a failed suicide attempt are often experienced by the older person, sometimes resulting in further attempts.

### Implications for future practice and research

It is notable that from this review that self-harming behaviors such as suicide attempts in older people is becoming a significant issue especially where in some countries the elderly suicide rate extends beyond 30% of all total reported suicides. It is also evident that the reasons for suicidal behaviors are multifactorial given the complex situations the older person finds themselves, especially within an Asian context. It is also important to note, that as this review is focused on studies undertaken in Asian countries, anecdotal evidence is now suggesting that older Asian populations residing in western countries are also seeing an increase in suicide attempts among this age group. Therefore, this is suggestive of a growing cultural problem internationally and not only isolated to older people in general. This is now being attributed to rising health care costs, increasing frailty from worsening chronic disease, and the erosion of traditional family values that are increasingly putting pressure on the older person to take their own life, if only to end their own physical suffering, but to also remove the perceived burden from children intent on living their own lives. As some of the triggers the older person is exposed to, like those mentioned above, are significant identifiers for suicidal thoughts and behaviors, the implications for clinical practice could include:

▪ Implementing a pre-discharge screening program to alert healthcare professionals to those older people presenting with pre-suicidal triggering factors, such as the expression of loneliness, feelings of desolation, despair and depression;
▪ Knowing the older person is at increased risk of repeated attempts, developing and promoting effective discipline specific after care interventions and support programs aimed at maintaining a sense of self-dignity and increased mindfulness.

It is noteworthy that there are few qualitative studies investigating suicidal ideation of older Asian people. To provide more insights, the implications for future healthcare research could include:

▪ Undertaking systematic and comprehensive reviews that includes literature presented in different languages, which would provide a more inclusive representation of elder suicidal ideation;
▪ More extensive qualitative research, such as the lived-experience or ethnographic studies of suicide ideation that provide greater insight into those pre-morbid thoughts prior to ideation such as the meaning of loneliness, desolation or despair;
▪ Qualitative studies that explore the impact of religion, filial piety, and gender has in supporting recovery from a suicide attempt in the older person.

## Conclusion

The growing trend of older people participating in suicidal behaviors is becoming a major health concern in some Asian countries. Rising healthcare costs, poverty, social isolation and a break with traditional Asian family values means that for some older people their children see them as a financial burden. This could be as a result of an increase in medical technology, globalization and wealth enhancement, which means that older people are living longer and are seen as a financial liability in the eyes of their children because of worsening chronic disease and increasing frailty. The result is a proportion of the older Asian population now seeing their sense of self-worth within the family unit is becoming increasingly eroded. It therefore comes as no surprise that when faced mounting opposition to their presence, suicide becomes the only viable solution to incredibly stressful situations. However, it must be stressed that not all older people contemplate this scenario, but instead live a productive post-retirement life with children who venerate the wealth of experience they bring. Yet sadly, there are those older people who feel there is very little scope for living a fulfilling life into old age.

## Author contributions

MC and HM: conceptualization, supervision, analysis, validation, and review and editing. HC, YC, KC, TC, TL, PT, and CT: investigation, data curation, analysis, and writing original draft. All authors contributed to the article and approved the submitted version.

## Conflict of interest

The authors declare that the research was conducted in the absence of any commercial or financial relationships that could be construed as a potential conflict of interest.

## Publisher’s note

All claims expressed in this article are solely those of the authors and do not necessarily represent those of their affiliated organizations, or those of the publisher, the editors and the reviewers. Any product that may be evaluated in this article, or claim that may be made by its manufacturer, is not guaranteed or endorsed by the publisher.

## References

[1] Fassberg MM Van Orden KA Duberstein P Erlangsen A Lapierre S Bodner E. A systematic review of social factor and suicidal behaviour in older adulthood. Int J Environ Res Public Health. (2012) 9:722–5. doi: 10.3390/ijerph9030722, PMID: 22690159 PMC3367273

[2] Hultcrantz M Svensson T Derolf AR Kristinsson SY Ekbom A Granath F. Incidence and risk factors for suicide and attempted suicide following a diagnosis of haematological malignancy. Cancer Med. (2015) 4:147–4. doi: 10.1002/cam4.316, PMID: 25155101 PMC4312128

[3] Webb RT Kontopantelis E Doran T Qin P Creed F Kapur N. Suicide risk in primary care patients with major physical diseases: a case-control study. Arch Gen Psychiatry. (2012) 69:256–4. doi: 10.1001/archgenpsychiatry.2011.1561, PMID: 22393218

[4] Durkheim É. Le suicide In: J Spalding T Simpson G, editors. Suicide: a study in sociology. London: Routledge & Kegan Paul (1897). 39–53.

[5] Leenaars AA. Psychotherapy with suicidal people: a person-centred approach. Chichester, John Wiley & Sons, Ltd: (2014).

[6] Troya M Babatunde O Polidano K Bartlam B McCloskey E Dikomitis L. Self-harm in older adults: systematic review. Br J Psychiatry. (2019a) 214:186–13. doi: 10.1192/bjp.2019.11 30789112

[7] Troya MI Dikomitis L Babatunde OO Bartlam B Chew-Graham CA. Understanding self-harm in older adults: a qualitative study. EClinicalMedicine. (2019b) 12:52–61. doi: 10.1016/j.eclinm.2019.06.002 31388663 PMC6677649

[8] National Institute for Health and Clinical Excellence. Self-harm in over 80s: long-term management. Clinical guideline [CG133]. London: National Institute for Health and Clinical Excellence (2011).

[9] Kim HK Ahn JS Kim H Cha YS Lee J Kim MH. Sociodemographic and clinical characteristics of old-old suicide attempters compared with young-old and middle-aged attempters. Int J Geriatr Psychiatry. (2018) 33:1717–26. doi: 10.1002/gps.4976, PMID: 30264415

[10] Chen Y-Y Chien Chang K Yousef S Yip PSF. Suicide in Asia: opportunities and challenges. Epidemiologic Reviews. (2012) 34:129–144. doi: 10.1093/epirev/mxr025 22158651

[11] Wand APF Peisah C Draper B Brodaty H. Understanding self-harm in older people: a systematic review of qualitative studies. Aging Ment Health. (2018a) 22:289–8. doi: 10.1080/13607863.2017.1304522, PMID: 28326821

[12] Liu Y-Y Wang X-T Qiu H-M Xu A-Q Jia C-X. Functional and dysfunctional impulsivity and attempted suicide in rural China: a paired case-control study. Psychiatry Res. (2017) 253:22–7. doi: 10.1016/j.psychres.2017.03.025, PMID: 28319788

[13] He J Ouyang F Qiu D Li L Li Y Xiao S. Time Trends and Predictions of suicide mortality for people aged 70 -years and over from 1990 to 2030 based on the Global Burden of Disease Study 2017. Frontiers in Psychiatry. (2021) 12:21343. doi: 10.3389/fpsyt.2021.721343 PMC8502866 34646174

[14] Tsoh J Chiu HFK Duberstein PR Chan SSM Chi I Yip PSF. Attempted suicide in elderly Chinese persons: a multigroup controlled study. J Geriatr Psychiatry. (2005) 13:562–1. doi: 10.1097/00019442-200507000-00004, PMID: 16009732

[15] Kim SY Kim MH Kawachi I Cho Y. Comparative epidemiology of suicide in South Korea and Japan: effects of age, gender and suicide methods. Crisis. (2011) 32:5–14. doi: 10.1027/0227-5910/a000046, PMID: 21371965

[16] Park BCB Lester D. Suicide in Asia: Causes and prevention. South Korea In: Yip PSF, editor. (Hong Kong, China: Hong Kong University Press) (2008)

[17] Pritchard C Baldwin DS. The one-child policy and parent-child relationships: a comparison of one-child with multiple-child families in China. Int J Sociol Soc Policy. (2002) 105:271–275. doi: 10.1034/j.1600-0447.2002.1014.x

[18] Moore SL. A phenomenological study of meaning in life in suicidal older adults. Arch Psychiatr Nurs. (1997) 11:29–36. doi: 10.1016/S0883-9417(97)80047-7, PMID: 9046641

[19] Kim OS Sok SR. Life experiences of elderly people with suicide ideation at the long term care hospitals in South Korea. International Journal of Nursing Practice. (2017) 23:e12597. doi: 10.1111/ijn.12597 28980749

[20] Kim Y. Understanding the life experiences of older adults in Korea following a suicide attempt. Qual Health Res. (2014) 24:1391–9. doi: 10.1177/1049732314547643, PMID: 25147216

[21] Murray J Banerjee S Byng R Tylee A Bhugra D Macdonald A. Primary care professionals’ perceptions of depression in older people: a qualitative study. Soc Sci Med. (2006) 63:1363–73. doi: 10.1016/j.socscimed.2006.03.037 16698157

[22] Bai X Zhou L Mo Q Jia C Ma Z. Understanding the reasons for suicide among older adults in rural China using in-depth interviews. Crisis J Crisis Interv Suic Prevent. (2021) 43:391–7. doi: 10.1027/0227-5910/a000799, PMID: 34406810

[23] Huang LB Tsai YF Liu CY Chen YJ. Influencing factors of suicidal ideation among older adults. Int J Ment Health Nurs. (2017) 26:191–9. doi: 10.1111/inm.12247, PMID: 27452945

[24] Lee SH Tsai YF Chen CY Huang LB. Triggers of suicide ideation and protective factors of actually executing suicide among first onset cases in older psychiatric outpatients: a qualitative study. BMC Psychiatry. (2014) 14:269. doi: 10.1186/s12888-014-0269-9, PMID: 25403893 PMC4237773

[25] Tsai YF Wong TKS Ku YC Liu WC. Reasons for living among older male Chinese residents of vetrans homes. J Adv Nurs. (2011) 68:1978–87. doi: 10.1111/j.1365-2648.2011.05884.x, PMID: 22103692

[26] Bonnewyn A Shah A Bruffaerts R Schoevaerts K Rober P Van Parys H. Reflections of older adults on the process preceding their suicide attempt: a qualitative approach. Death Stud. (2014) 38:612–8. doi: 10.1080/07481187.2013.835753, PMID: 24521397

[27] Lockwood C Porritt K Munn Z Rittenmeyer L Salmond S Bjerrum M. JBI Manual for Evidence Synthesis. JBI, 2020. In: Aromataris E Munn Z, editors. Systematic reviews of qualitative evidence. (2020) Available from: https://synthesismanual.jbi.global

[28] Tong A Sainsbury P Craig J. Consolidated criteria for reporting qualitative research (COREQ): a 32-item checklist for interviews and focus groups. Int J Qual Health Care. (2007) 19:349–7. doi: 10.1093/intqhc/mzm042, PMID: 17872937

[29] Moher D Liberati A Tetzlaff J Altman DG. Preferred reporting items for systematic reviews and Meta-analyses: the PRISMA statement. PLoS Med. (2009) 6:e1000097. doi: 10.1371/journal.pmed1000097, PMID: 19621072 PMC2707599

[30] Munn Z Poritt K Lockwood C Aromatris E Pearson A. Establishing confidence in the output of qualitative research synthesis: the ConQual approach. BMC Med Res Methodol. (2014) 14:108. doi: 10.1186/1471-2288-14-108, PMID: 25927294 PMC4190351

[31] McArthur A Klugarova J Yan H Florescu S. Suicide in Asia: opportunities and challenges. Systematic reviews of text and opinion. In: Aromataris E Munn Z, editors. (2020) Available from: https://synthesismanual.jbi.global

[32] Ku YC Tsai YF Lin YC Lin YP. Suicide experiences among institutionalised older veterans in Taiwan. Gerontologist. (2009) 49:746–54. doi: 10.1093/geront/gnp114, PMID: 19597056

[33] Lyndon N Azman H Rose RAC Jali MFM. Sociological narrative of suicidal behaviour among older people. Clinical Interventions in Aging. (2021) 16:1379–1392. doi: 10.1080/13607863.2017.1304522, PMID: 34290500 PMC8289311

[34] Li X Xiao Z Xiao S. Suicide among the elderly in mainland China. Psychogeriatrics Off J Jap Psychogeriatric Soc. (2009) 9:62–6. doi: 10.1111/j.1479-8301.2009.00269.x 19604327

[35] Cheung G Foster G de Beer W Gee S Hawkes T Rimkeit S. Predictors for repeat self-harm and suicide among older people within 12 months of a self-harm presentation. Int Psychogeriatr. (2017) 29:1237–45. doi: 10.1017/S1041610217000308, PMID: 28349860

[36] Crocker L Clare L Evans K. Giving up or finding a solution? The experience of attempted suicide in later life. Aging Men Health. (2006) 10:47. doi: 10.1080/13607860600640905 17050093

[37] Wiktorsson S Runeson B Skoog I Östling S Waern M. Attempted suicide in the elderly: characteristics of suicide attempters 70 years and older and a general population comparison group. Am J Geriatr Psychiatry. (2010) 18:57–67. doi: 10.1097/JGP.0b013e3181bd1c13, PMID: 20094019

[38] Du P Qu JY. Filial piety of children as perceived by aging parents in China: trends and determinants. Popul Res. (2013) 37:30. (in Chinese)

[39] Xu HL. Epidemiological study on committed suicide among the elderly in some urban and rural areas of Hunan Province. Chin Ment Health J. (2000) 14:121–4.

[40] Chow ENL Zhao SM. The one-child policy and parent-child relationships: a comparison of one-child with multiple-child families in China. Int J Sociol Soc Policy. (1996) 16:35–62. doi: 10.1108/eb013285

[41] De Sousa GS Perrelli JGA Botelho ES. Nursing diagnosis for risk of suicide in elderly: integrative review. Rev Gaucha Enferm. (2018) 39:e20170120. doi: 10.1590/1983-1447.2018.2017-0120, PMID: 30088601

[42] Lutzman M Sommerfeld E Ben-David S. Loneliness and social integration as mediators between physical pain and suicidal ideation among elderly men. Int Psychogeriatr. (2021) 33:453–9. doi: 10.1017/S104161022000112X, PMID: 32641182

[43] Wang J Ho E Au P Cheung G. Late-life suicide in Asian people living in New Zealand: a qualitative study of coronial records: late-life suicide in Asian people in NZ. Psychogeriatrics. (2018b) 18:259–7. doi: 10.1111/psyg.12318, PMID: 30133942

[44] Gutierrez DMD Sousa ABL Grubits S. Suicidal ideation and attempted suicide in elderly people - subjective experiences. Ciência Saude Coletiva. (2015) 20:1731–40. doi: 10.1590/1413-81232015206.02242015, PMID: 26060951

[45] Flett GL Heisel MJ. Aging and feeling valued versus expendable during the COVID-19 pandemic and beyond: a review and commentary of why mattering is fundamental to the health and well-being of older adults. Int J Ment Heal Addict. (2021) 19:2443–69. doi: 10.1007/s11469-020-00339-4, PMID: 32837430 PMC7295320

[46] Gruenewald TL Karlamangla AS Greendale GA Singer BH Seeman TE. Feelings of usefulness to others, disability, and mortality in older adults: the MacArthur study of successful aging. J Gerontol Psychol Sci. (2007) 62:P28–37. doi: 10.1093/geronb/62.1.P28 17284554

[47] Flett GL. An introduction, review, and conceptual analysis of mattering as an essential construct and an essential way of life. J Psychoeduc Assess. (2022) 40:3–36. doi: 10.1177/07342829211057640

[48] Dixon AL. Mattering in the later years: older adults’ experiences of mattering to others, purpose in life, depression, and wellness. Adultspan J. (2007) 6:83–95. doi: 10.1002/j.2161-0029.2007.tb00034.x

[49] AshaRani PV Lai D Koh J Subramaniam M. Purpose in life in older adults: a systematic review on conceptualization, measures, and determinants. Int J Environ Res Public Health. (2022) 19:1–25. doi: 10.3390/ijerph19105860 PMC9141815 35627396

